# Pediatric Acute Leukemias: Epidemiological, Clinical Features, and Diagnostic Contribution of Flow Cytometry in a Resource-Limited Setting

**DOI:** 10.7759/cureus.113650

**Published:** 2026-07-30

**Authors:** Ismail Regragui, Hassane Mamad, Jalila Zirar, Yasmine Sbay, Mohamed Ifleh, Souad Benkirane, Azlarab Masrar

**Affiliations:** 1 Central Laboratory of Hematology, Ibn Sina Hospital, University Mohammed V of Medicine, Rabat, MAR

**Keywords:** acute leukemia, bal, children, flow cytometry, morocco

## Abstract

Introduction: Acute leukemias are the most common hematologic malignancies in children, but detailed epidemiological and immunophenotypic data are limited in Morocco. A better characterization of these diseases is needed to optimize diagnostic strategies.

Objective: To describe the epidemiological, clinical, and biological features of pediatric acute leukemias and to evaluate the diagnostic contribution of flow cytometry in a Moroccan university hospital center, based on available diagnostic tools.

Methods: We conducted a retrospective, single-center study at the hematology laboratory of Ibn Sina University Hospital, Rabat, including all children under 15 years diagnosed with acute leukemia between January 1, 2022, and January 1, 2024. Diagnosis was based on bone marrow cytology, cytochemistry, and flow cytometry. Cases were classified according to the World Health Organization (WHO) 2008 classification based on the available diagnostic tools, as cytogenetic and molecular studies were not available in our setting. Cytochemical staining for myeloperoxidase (MPO) was systematically performed. Positivity thresholds for flow cytometry followed European Group for the Immunological Characterization of Leukemias (EGIL) recommendations. Statistical analyses used Fisher's exact and Kruskal-Wallis tests, with p < 0.05 considered significant.

Results: A total of 106 children with a mean age of 6.9 years were included. Acute lymphoblastic leukemia (ALL) accounted for 81.1% (n = 86), acute myeloid leukemia (AML) for 16.0% (n = 17), and biphenotypic acute leukemia (BAL) for 2.8% (n = 3). Three cases of Burkitt lymphoma were identified and excluded from the ALL group. The overall male-to-female ratio was 1.86 (69 boys, 37 girls), with no significant difference between subtypes (p = 0.46). Subtype distribution varied significantly with age (p = 0.02). The main clinical features were tumor syndrome (84.0%), fever (67.0%), and hemorrhagic syndrome (50.9%). Fever was significantly more frequent in B-ALL than in other subtypes (p < 0.001), as was hemorrhagic syndrome (p = 0.03). Significant differences between subtypes were also observed for leukopenia (p = 0.04), leukocytosis (p = 0.003), pancytopenia (p = 0.03), and major hyperleukocytosis (>100 × 10^9^/L, p < 0.001). Hypercellular marrow was found in 84.4% of cases, with a median blast count of 88% (interquartile range, IQR: 78-92), significantly higher in ALL than in AML (p = 0.008). Immunophenotyping allowed reclassification of six of the 95 MPO-negative suspected ALL cases: four were reclassified as acute myeloid leukemia with minimal differentiation and two as BAL. In addition, one BAL was identified among the 14 MPO-positive cases, raising the total number of BAL to three.

Conclusions: Flow cytometry is a valuable tool for the diagnosis of pediatric acute leukemias in resource-limited settings, contributing to the classification of ALL, identification of acute myeloid leukemia with minimal differentiation, and diagnosis of BAL based on cytology, cytochemistry, and immunophenotyping. The high frequency of aberrant antigen expression supports the need for a standardized diagnostic algorithm combining cytology, cytochemistry, and immunophenotyping. Larger prospective studies with cytogenetics and follow-up are needed to refine prognostic value.

## Introduction

Acute leukemias (ALs) are the most frequent hematologic malignancies in children, accounting for approximately 30% of all pediatric cancers [1]. The annual incidence is estimated at three to five cases per 100,000 children in Western countries, with a peak incidence between two and five years of age [2]. Acute lymphoblastic leukemias (ALLs) clearly predominate, representing 75-80% of cases, whereas acute myeloid leukemias (AMLs) account for 15-20%, and biphenotypic acute leukemias (BALs) represent less than 5% [2].

The diagnosis of AL is suggested by the presence of blast cells on a peripheral blood or bone marrow smear, in a setting of hyperleukocytosis or, conversely, peripheral cytopenia, with or without a tumor syndrome or other suggestive signs [3]. Although the cytological approach retains its full value, immunophenotyping by flow cytometry has become an essential step in the diagnosis of AL, serving as an indispensable complement to morphological examination. It allows confirmation of the cell lineage involved in the leukemic process and determination of the stage of blast differentiation arrest [4]. Specific techniques such as cytogenetics and molecular biology, despite their costs and limited availability in some hospital centers, play a crucial role in diagnostic confirmation, particularly when immunophenotyping is inconclusive or for the identification of rare molecular abnormalities. They are also essential for treatment initiation and for the monitoring of minimal residual disease [5].

In Morocco, detailed data on the epidemiological and biological profile of pediatric acute leukemias remain limited. A better characterization of these diseases in the national context is necessary to optimize diagnostic strategies and adapt therapeutic management. The present study aims to describe the clinical and immunophenotypic patterns of pediatric acute leukemias and to evaluate the diagnostic contribution of flow cytometry in a Moroccan University Hospital Center, based on the available diagnostic tools. We recognize that flow cytometry, while essential for lineage assignment, is not sufficient for a complete World Health Organization (WHO) 5th edition classification, which requires cytogenetic and molecular data. For this reason, and given the limited resources in our setting, we based our diagnostic classification on the WHO 2008 criteria, which rely on morphology, cytochemistry, and immunophenotyping.

## Materials and methods

This was a retrospective, descriptive, single-center study conducted at the Hematology Laboratory of Ibn Sina University Hospital Center in Rabat, Morocco, including all pediatric acute leukemia cases diagnosed consecutively over a 24-month period (January 1, 2022-January 1, 2024). Of 121 patients initially screened, 12 were excluded due to incomplete medical records or absence of immunophenotyping confirmation, leaving 109 patients for final analysis. Data were retrospectively extracted from patients' medical records and laboratory reports, collecting demographic data (age at diagnosis, sex), initial clinical data (tumor syndrome defined as lymphadenopathy, hepatomegaly, splenomegaly, or mediastinal mass; fever >38°C; hemorrhagic syndrome), and initial biological data. The Ethics Committee of Ibn Sina University Hospital confirmed that formal approval was not required for this retrospective study using anonymized data.

Complete blood count was performed on a Beckman Coulter analyzer, recording hemoglobin level (g/dL), platelet count (×10^9^/L), and white blood cell count (×10^9^/L). Anemia was defined according to World Health Organization (WHO) criteria as hemoglobin below age-specific thresholds; thrombocytopenia as platelet count <150 × 10^9^/L; leukopenia as white blood cell count <4 × 10^9^/L; leukocytosis as white blood cell count >12 × 10^9^/L; and pancytopenia as the association of anemia, thrombocytopenia, and leukopenia. Bone marrow smears stained with May-Grünwald-Giemsa (MGG) were examined cytologically. The diagnosis of acute leukemia was established according to the WHO 2008 classification when blast cells exceeded 20% of nucleated marrow cells; blast percentage was assessed by aspirate morphology on MGG smears. Hemodilute samples were not systematically excluded, but two senior biologists reviewed all samples, and only those considered adequate were included. Myeloperoxidase (MPO) cytochemistry was systematically performed as an initial screening tool (positivity threshold: 3% of blasts) to guide diagnostic orientation and select the appropriate flow cytometry panel. At the same time, definitive lineage assignment was established by immunophenotyping.

Immunophenotyping was performed on bone marrow samples collected in heparin or ethylenediaminetetraacetic acid (EDTA) at diagnosis, using a Sysmex XF-1600 flow cytometer (Sysmex Corporation, Japan) with a PS-10 sample preparation system, following internationally recognized recommendations. For each sample, 10,000 events per tube were acquired from samples with viability >85%; compensation was performed automatically using single-stained controls, and daily quality control was ensured using commercial calibration beads according to the manufacturer's recommendations. Blasts were identified using a sequential gating strategy: cells were first selected based on forward scatter (FSC)/side scatter (SSC) to exclude debris, doublets were excluded using FSC-H/FSC-A and SSC-H/SSC-A, blasts were then identified based on CD45-dim/SSC-low characteristics, and lineage assignment was finally performed using specific markers. Cells were labeled with a standardized panel of fluorochrome-conjugated monoclonal antibodies including myeloid markers (CD13, CD33, CD117, intracytoplasmic MPO), B-lymphoid markers (CD19, CD10, intracytoplasmic CD79a, CD22, TdT), T-lymphoid markers (intracytoplasmic CD3, CD7, CD5, CD4, CD8), and additional markers (CD34, HLA-DR). Positivity thresholds were set at ≥20% of blasts for membrane markers and ≥10% for cytoplasmic and intranuclear markers, in accordance with recommendations of the European Group for the Immunological Characterization of Leukemias (EGIL). Aberrant antigen expression was assessed exclusively on the blast population, excluding background lymphoid and myeloid cells by gating.

For the diagnosis of acute leukemias, we used a complementary approach combining WHO 2008 criteria for overall diagnostic classification and EGIL scoring for immunophenotypic lineage assignment. A case was defined as biphenotypic acute leukemia (BAL) when it reached a score of at least two points for two separate lineages, according to EGIL criteria. All cytological, cytochemical, and immunophenotypic examinations were reviewed and validated by two senior medical biologists, and the final classification was established by integrating morphological, cytochemical, and immunophenotypic data. We acknowledge that a complete WHO 5th edition classification requires cytogenetic and molecular data, which were not available in our setting; our diagnostic approach therefore reflects the reality of resource-limited laboratories where diagnosis relies primarily on cytology, cytochemistry, and immunophenotyping. Additionally, mature B-cell neoplasms, such as Burkitt lymphoma/leukemia, were excluded from the B-ALL group and analyzed separately, in accordance with WHO 2008 criteria. Statistical analysis was performed using IBM SPSS Statistics version 25.0 (IBM Corp., Armonk, New York, USA). Qualitative variables were expressed as counts and percentages, and quantitative variables as mean ± standard deviation or median with interquartile range depending on distribution. Comparisons of qualitative variables used Fisher's exact test, and quantitative variables used the Kruskal-Wallis test, with statistical significance set at p < 0.05.

## Results

According to the classification established by immunophenotyping following international recommendations, the distribution of the 109 initially suspected acute leukemia cases showed a clear predominance of ALL, which accounted for 81.1% of diagnoses (n = 86), with a majority of B-ALL (65.1%, n = 69) versus 16.0% (n = 17) for T-ALL. AML accounted for 16.0% (n = 17) and BAL for 2.8% (n = 3) of cases. Three cases of Burkitt lymphoma were identified and excluded from the ALL group, in accordance with WHO 2008 criteria. The mean age was 6.9 ± 3.8 years (range: 3 days to 15 years). The overall male-to-female ratio was 1.86 (69 boys and 37 girls) after exclusion of the three Burkitt cases. In B-ALL, there were 42 boys and 27 girls (sex ratio 1.56); in T-ALL, 14 boys and 3 girls (sex ratio 4.67); in AML, 10 boys and 7 girls (sex ratio 1.43); and in BAL, 3 boys and 0 girls, with no significant difference between leukemic subtypes (p = 0.46). The distribution of leukemia subtypes varied significantly with age (p = 0.02). In infants under one year of age, B-ALL was the predominant subtype, accounting for 88.2% of cases (n = 15 out of 17), followed by T-ALL and AML (5.9% each). In the 1-10-year age group, B-ALL remained the most frequent subtype (65.7%, n = 48 out of 73), ahead of T-ALL (17.8%, n = 13), AML (13.7%, n = 10), and BAL (2.8%, n = 2). In the 10-15-year age group, B-ALL and AML were equally represented (37.5% each, n = 6), followed by T-ALL (18.75%, n = 3) and one case of BAL (6.25%). Despite their small numbers, T-ALL and AML were present in all age groups. All these demographic and subtype distribution data are summarized in Table 1.

**Table 1:** Demographic characteristics of the cohort. n: number of patients; M/F: number of male/number of female patients; B-ALL: B-cell acute lymphoblastic leukemia; T-ALL: T-cell acute lymphoblastic leukemia; AML: acute myeloid leukemia; BAL: biphenotypic acute leukemia.

Age group	n	B-ALL (M/F)	T-ALL (M/F)	AML (M/F)	BAL (M/F)	Total M/F
<1 Year	17	15 (11/4)	1 (1/0)	1 (0/1)	0	12/5
1-10 Years	73	48 (28/20)	13 (11/2)	10 (6/4)	2 (2/0)	47/26
10-15 Years	16	6 (3/3)	3 (2/1)	6 (4/2)	1 (1/0)	10/6
Total	106	69 (42/27)	17 (14/3)	17 (10/7)	3 (3/0)	69/37

Clinical signs were recorded in 103 patients (97.2% of the 106 included patients). The main clinical manifestations were tumor syndrome (84.0%, n = 89), fever (67.0%, n = 71), and hemorrhagic syndrome (50.9%, n = 54). Emergency situations related to hyperleukocytosis exceeding 100 × 10^9^/L were reported in 18 patients (17.0% of the cohort). Statistical analysis showed a highly significant difference in fever among the four subtypes (p < 0.001), a significant difference in hemorrhagic syndrome (p = 0.03), and no significant difference in tumor syndrome (p = 0.08). Table 2 details the prevalence of these manifestations according to leukemia subtype (B-ALL, T-ALL, AML, and BAL).

**Table 2:** Prevalence of main clinical manifestations. n: number of patients; ×10^9^/L: ×10^9^ per liter; B-ALL: B-cell acute lymphoblastic leukemia; T-ALL: T-cell acute lymphoblastic leukemia; AML: acute myeloid leukemia; BAL: biphenotypic acute leukemia. Tumor syndrome was defined as the presence of at least one of the following: lymphadenopathy, hepatomegaly, splenomegaly, or mediastinal mass. Fever was recorded as a clinical sign at presentation regardless of its underlying cause.

Clinical feature	B-ALL (n = 69)	T-ALL (n = 17)	AML (n = 17)	BAL (n = 3)	Total (N = 106)
Tumor syndrome, n (%)	62 (89.9%)	13 (76.5%)	11 (64.7%)	3 (100%)	89 (84.0%)
Fever, n (%)	59 (85.5%)	5 (29.4%)	5 (29.4%)	2 (66.7%)	71 (67.0%)
Hemorrhagic syndrome, n (%)	41 (59.4%)	4 (23.5%)	7 (41.2%)	2 (66.7%)	54 (50.9%)
Hyperleukocytosis >100 × 10^9^/L, n (%)	5 (7.2%)	10 (58.8%)	3 (17.6%)	0 (0%)	18 (17.0%)

Complete blood count analysis revealed frequent abnormalities. Anemia was present in 93.4% of cases (99/106), with a lower mean hemoglobin level in AML (6.70 g/dL) compared with B-ALL (8.15 g/dL), T-ALL (8.20 g/dL), and BAL (7.50 g/dL). Thrombocytopenia was observed in 78.3% of cases (83/106), with a lower mean platelet count in AML (21.0 × 10^9^/L) than in B-ALL (44.2 × 10^9^/L), T-ALL (67.6 × 10^9^/L), and BAL (35.0 × 10^9^/L). Regarding the leukocyte profile, leukopenia was noted in 30.2% of cases (32/106), more frequent in B-ALL, whereas leukocytosis was observed in 52.8% of cases (56/106), proportionally more frequent in T-ALL and AML (76.5% each) than in B-ALL and BAL. Pancytopenia was found in 21.7% of patients (23/106): 22 cases of B-ALL and one case of T-ALL. In terms of frequency, statistical analysis showed a significant difference between the four subtypes for leukopenia (p = 0.04), leukocytosis (p = 0.003), and pancytopenia (p = 0.03), whereas no significant difference was observed for anemia (p = 0.92) or thrombocytopenia (p = 0.31). Regarding major hyperleukocytosis (>100 × 10^9^/L), the difference between subtypes was highly significant (p < 0.001), with a marked predominance in T-ALL (58.8%). These parameters are detailed according to subtype in Table 3.

**Table 3:** Comparison of hematological profiles by leukemia subtype. n: number of patients; Hb: hemoglobin; plt: platelets; WBC: white blood cells; 10^9^/L: × 10^9^ per liter ; B-ALL: B-cell acute lymphoblastic leukemia; T-ALL: T-cell acute lymphoblastic leukemia; AML: acute myeloid leukemia; BAL: biphenotypic acute leukemia. Mean values for hemoglobin, platelets, and WBC count were calculated only among patients with the corresponding abnormality (anemia, thrombocytopenia, leukopenia, etc.), as indicated by the n value for each parameter. This table is descriptive. Corresponding p-values are provided in the preceding paragraph of the Results section.

Parameter	B-ALL (n = 69)	T-ALL (n = 17)	AML (n = 17)	BAL (n = 3)	Total (N = 106)
Anemia, n (mean Hb in g/dL)	64 (8.15)	16 (8.20)	16 (6.70)	3 (7.50)	99
Thrombocytopenia, n (mean platelets × 10⁹/L)	53 (44.20)	12 (67.60)	16 (21.00)	2 (35.00)	83
Thrombocytosis, n (mean platelets × 10⁹/L)	6 (553)	1 (650)	0	0	7
Leukopenia, n (mean WBC × 10⁹/L)	27 (2.50)	2 (1.82)	2 (2.00)	1 (2.20)	32
Leukocytosis, n (mean WBC × 10⁹/L)	29 (74.95)	13 (223.90)	13 (102.00)	1 (65.00)	56
Pancytopenia, n	22	1	0	0	23

Bone marrow examination revealed hypercellular marrow in 84.4% of cases, with massive blast infiltration. The median blast percentage was 88% (interquartile range, IQR: 78-92). This infiltration was particularly high and homogeneous in ALL (median 88%, IQR: 82-93), whereas it was lower and more heterogeneous in AML (median 77%, IQR: 57-84). Statistical analysis showed a significant difference in blast percentages between ALL and AML (p = 0.008). Systematic myeloperoxidase (MPO) cytochemistry was positive in 14 cases (12.8%) and negative in 95 cases (87.2%), guiding the immunophenotyping strategy. Analysis of the 95 MPO-negative cases allowed precise lineage determination: 86 cases were confirmed as ALL, comprising 69 B-ALL and 17 T-ALL. Three cases were identified as Burkitt lymphoma based on immunophenotyping and were not grouped with B-ALL, in accordance with WHO 2008 criteria. Among the remaining cases, four were classified as acute myeloid leukemia with minimal differentiation, and two were diagnosed as BAL. Among the 14 MPO-positive cases, immunophenotyping confirmed a myeloid phenotype in 13 cases, while the 14th case proved to be a BAL, bringing the total number of BAL to 3.

In B-ALL cases, CD19 was expressed in 97% of cases, while CD10, intracytoplasmic CD79a, TdT, and HLA-DR were consistently positive (100% each). CD34 was positive in 54% of cases. The 17 T-ALL cases expressed intracytoplasmic CD3 (100%), CD7 (100%), and CD5 (88%), with CD4/CD8 co-expression observed in only four cases. In AML (n = 17), the phenotype was dominated by expression of CD33 (88%), MPO detected by flow cytometry (88%), CD117 (88%), and CD13 (82%). CD34 was expressed in 65% of AML cases. The three BAL cases met EGIL criteria for biphenotypic acute leukemia, each showing co-expression of markers from two different lineages, with a total score of at least two points for each lineage.

Aberrant antigen expression was frequently observed. In AML (n = 17), expression of the T-lymphoid marker CD7 was noted in 76.5% of cases (13/17). Expression of B-lymphoid markers was also common: CD19 in 70.6% (12/17) and CD10 in 82.4% (14/17) of cases. Conversely, in B-ALL (n = 69), expression of myeloid markers was notable: CD33 was expressed in 55.1% of cases (38/69), and CD13 in 53.6% of cases (37/69). In addition, the T-lymphoid marker CD7 was aberrantly expressed in 73.9% of B-ALL cases (51/69).

## Discussion

Acute leukemias are the most frequent hematologic malignancies in children, with a well-established worldwide predominance of ALL over AML [1]. Our series of 106 cases confirms this trend (81.1% ALL, 16.0% AML, 2.8% BAL), a distribution comparable to that reported in the international and regional literature and close to that reported by Doumbia et al. in the same center [6-8]. Three cases of Burkitt lymphoma were identified and excluded from the ALL group, in accordance with WHO 2008 criteria.

Our study population had a mean age of 6.9 ± 3.8 years (range: 3 days to 15 years), with a marked peak incidence in children aged 1 to 10 years (66.9% of cases). These values are comparable to those reported by Jamal et al. in a large series of 1379 pediatric patients (mean age 7.4 ± 4.3 years, peak incidence in the 1-10-year age group at 70.7%) [6]. Similarly, our overall male-to-female ratio of 1.86 is close to the ratio of 1.75 reported by Jamal et al. By subtype, the sex ratios were 1.56 for B-ALL, 4.67 for T-ALL, and 1.43 for AML, all in favor of boys, comparable to those reported by the same authors (1.5, 3.8, and 1.5, respectively). The distribution of leukemia subtypes varied significantly with age (p = 0.02). In infants under one year of age, B-ALL was the predominant subtype, accounting for 88.2% of cases (n = 15 out of 17). In the 1-10-year age group, B-ALL remained the most frequent (65.7%, n = 48), ahead of T-ALL (17.8%, n = 13), AML (13.7%, n = 10), and BAL (2.8%, n = 2). In children aged 10-15 years, B-ALL and AML were equally represented (37.5% each, n = 6), followed by T-ALL (18.75%, n = 3) and one case of BAL (6.25%). This age distribution is consistent with the data from Jamal et al., who reported a predominance of ALL in children aged 1-10 years (77.1%), an increase in the frequency of AML in those over 10 years of age (31.1%), and a decrease in the frequency of ALL in the same age group [6]. These results are also in agreement with those of Doumbia et al. [7]. The studies used for comparison employed similar diagnostic frameworks based on morphology, cytochemistry, and immunophenotyping, ensuring comparability of the data. All these results are summarized in Table 4, which compares our demographic profile and subtype distributions with those of the study by Jamal et al. [6].

**Table 4:** Demographic profile and subtype distribution. Comparison with the findings of Jamal et al. [6] n: number of patients; ALL: acute lymphoblastic leukemia; AML: acute myeloid leukemia; BAL: biphenotypic acute leukemia; M/F: male-to-female ratio.

Characteristic	Our study (n = 106)	Jamal et al. (n = 1379)
Mean age (years)	6.9 ± 3.8	7.4 ± 4.3
Peak incidence (1-10 years)	66.9%	70.7%
Overall M/F ratio	1.86	1.75
M/F ratio B-ALL	1.56	1.5
M/F ratio T-ALL	4.67	3.8
M/F ratio AML	1.43	1.5
ALL (%)	81.1%	77.2%
AML (%)	16.0%	21.2%
BAL (%)	2.8%	1.6%
ALL in 1-10 years (%)	83.5%	77.1%
AML in >10 years (%)	37.5%	33.2%

The symptoms of acute leukemia are mainly related to bone marrow and extramedullary infiltration by uncontrolled proliferating blasts. This infiltration frequently manifests as enlargement of the hematopoietic organs, including lymphadenopathy (cervical, inguinal, mediastinal, submandibular), splenomegaly, and hepatomegaly, leading to increased organ volume and functional impairment [9]. In our cohort, the initial manifestations were dominated by tumor syndrome (84.0%), fever (67.0%), and hemorrhagic syndrome (50.9%). This classic triad of bone marrow failure and blast proliferation is characteristic of pediatric acute leukemias [10]. However, the frequency of these manifestations varied according to the leukemia subtype. A notable difference concerned the prevalence of fever, which was very high in B-ALL (85.5%) compared with the other subtypes (approximately 30% each), with a highly significant difference (p < 0.001). Similarly, hemorrhagic syndrome was more frequent in B-ALL (59.4%) than in the other subtypes (p = 0.03). In contrast, no significant difference was observed for tumor syndrome between subtypes (p = 0.08). These findings should be interpreted with caution, as subgroup sizes, particularly BAL (n = 3), were very small. They are presented as descriptive observations rather than definitive conclusions.

Biologically, anemia and profound thrombocytopenia were almost constant across all leukemia subtypes, with no significant differences among them (p = 0.92 for anemia, p = 0.31 for thrombocytopenia). In contrast, the initial leukocyte profile showed significant heterogeneity according to subtype (p = 0.04 for leukopenia, p = 0.003 for leukocytosis). T-ALL and AML were frequently associated with hyperleukocytosis (76.5% each), whereas B-ALL was characterized by an almost equivalent distribution between leukocytosis (42.0%) and leukopenia (39.1%). The incidence of major hyperleukocytosis (>100 × 10^9^/L) was particularly high in T-ALL (58.8%) and, to a lesser extent, in AML (17.6%), with a highly significant difference between subtypes (p < 0.001). These results are comparable to those reported in the international literature, notably by Kittivisuit et al., who found a similar incidence of major hyperleukocytosis in T-ALL and AML [11]. Extreme hyperleukocytosis (>100 × 10^9^/L) has been described in the literature as a poor prognostic factor, whereas a leukocyte count below this threshold is classically associated with a more favorable prognosis [12]. However, no prognostic conclusions can be drawn from our study, as we did not have survival or outcome data. These p-values should be interpreted with caution, given the small size of certain subgroups.

Beyond peripheral blood abnormalities, bone marrow examination provided additional insights. It showed hypercellular marrow in 84.4% of cases, with massive blast infiltration throughout the cohort and a median blast percentage of 88% (IQR: 78-92). This infiltration was higher and more homogeneous in ALL (median 88%, IQR: 82-93) than in AML (median 77%, IQR: 57-84), with a statistically significant difference (p = 0.008). These values are comparable to those reported in other pediatric series, notably by Doumbia et al., who found a median blast percentage of 90.4% (IQR: 87-99) in ALL and 78% (IQR: 53-88) in AML [7].

In addition to cytological and cytochemical analysis, immunophenotyping provided critical diagnostic information. Among the 95 cases suspected of ALL based on morphology and negative MPO, 86 were confirmed as ALL (69 B-ALL and 17 T-ALL), while the remaining six cases were reclassified (four acute myeloid leukemia with minimal differentiation and two BAL). Three cases of Burkitt lymphoma were identified and excluded from the B-ALL group, in accordance with WHO 2008 criteria. This distinction is crucial because T-ALL carries a higher risk of early relapse, particularly medullary and neurological, justifying more intensive treatment [13]. It also guides therapeutic choice: B-ALL is particularly sensitive to asparaginase and high-dose methotrexate, whereas T-ALL relies more on anthracyclines [13]. Beyond lineage distinction, immunophenotyping was also decisive in confirming the diagnosis in the three suspected cases of Burkitt lymphoma, an entity distinct from common B-ALL, characterized by rapid tumor kinetics and requiring a specific, urgent therapeutic protocol [14]. Complementarily, immunophenotyping proved essential for the precise diagnosis of AML. Among the 14 MPO-positive cases, 13 were confirmed as AML and one case was identified as BAL. This latter case, along with the two BAL cases already diagnosed among MPO-negative cases, brought the total to three BAL cases in our cohort. Thus, our results emphasize, in agreement with the literature, that flow cytometry is an essential tool for the diagnosis of BAL by demonstrating concomitant expression of markers from two different lineages on the same blast clone, with a score of at least two points for each lineage, as defined by EGIL criteria [4,15].

The immunophenotypic profiles observed in our cohort reflect the major diagnostic features of acute leukemias described in the literature. In B-ALL (n = 69), the combination of strong CD19 expression (97%) and intracytoplasmic CD79a (100%), together with TdT and CD10 (100% of tested cases), constitutes a major diagnostic profile, widely described in the literature. In T-ALL (n = 17), the expression of intracytoplasmic CD3 and CD7, observed in 100% of cases each, is in perfect agreement with international standards. For AML (n = 17), our series confirms the high sensitivity of conventional markers, with predominant expression of CD33 (88%), MPO detected by flow cytometry (88%), CD117 (88%), and CD13 (82%). Of note, MPO detection by flow cytometry proved to be significantly more sensitive than cytochemistry, confirming its superiority for identifying myeloid lineages, particularly acute myeloid leukemia with minimal differentiation [16,17].

Aberrant expression of myeloid and lymphoid antigens is a well-documented phenomenon and has been described as a potential tool for minimal residual disease monitoring [18,19]. Our study highlights the high frequency of these aberrant expressions, the extent of which in our cohort is notable. In B-ALL, myeloid marker expression (CD13 in 53.6% and CD33 in 55.1%) is consistent with frequencies reported in other series, which generally range from 20% to 50% [20]. These results are also comparable to those of Moroccan studies: Bachir et al. found myeloid marker expression in 52.7% of ALL (57.4% of B-ALL and 33.9% of T-ALL), while Tlamçani et al. reported aberrant phenotypes in 49.4% of cases (52.9% of B-ALL and 30.8% of T-ALL), with a predominance of CD33 and CD13 myeloid markers [21,22]. Conversely, aberrant expression of lymphoid markers in AML was particularly frequent, affecting CD7 in 76% (13/17), CD10 in 82% (14/17), and CD19 in 70% (12/17) of cases. Although among the 13 CD7-positive AML patients in our series only 3 (23%) had major hyperleukocytosis (>100 × 10⁹/L), a study by Chang et al. demonstrated that CD7 positivity in AML is significantly associated with unfavorable clinical characteristics, including a white blood cell count above 100 × 10⁹/L, as well as a poorer prognosis, with significantly lower complete remission rates and overall survival compared with CD7-negative patients [23]. These very high percentages of aberrant markers observed in our study must be interpreted with caution. It is important to distinguish isolated aberrant expression of a single marker from true biphenotypic acute leukemia (BAL); only cases meeting EGIL criteria (score ≥2 for two separate lineages) were classified as BAL in our study. Nevertheless, they underscore the critical importance of knowing these aberrant profiles to avoid interpretation errors: a single marker is not sufficient to establish the diagnosis. It is the combined analysis of several specific antigens that allows distinguishing a genuine phenotypic ambiguity, as in the BAL cases in our series, from an isolated aberration without impact on the final classification. This fundamental principle fully justifies the systematic use of extended panels in flow cytometry for the initial diagnosis of acute leukemias [24,25].

Our study has several limitations. First, its retrospective and single-center nature may introduce selection bias and limit the generalizability of the results. Second, the absence of cytogenetic and molecular analyses prevents a full classification according to the WHO 5th edition and limits prognostic stratification and therapeutic guidance. Third, the small number of certain subtypes does not allow robust statistical analyses and may have overestimated the frequency of some aberrant expressions. In particular, findings related to BAL should be interpreted with caution and may not be generalizable. Fourth, due to the retrospective nature of the study, detailed immunophenotypic profiles, raw flow cytometry data, and representative plots were not available for verification, particularly for the reclassified cases (AML with minimal differentiation and BAL). Fifth, the lack of therapeutic follow-up and minimal residual disease monitoring makes it impossible to evaluate the relationship between aberrant antigen expression and adverse clinical outcomes, such as relapse or death. Prospective multicenter studies with standardized protocols, including cytogenetic and molecular analyses, larger cohorts, and long-term follow-up, are needed to overcome these limitations and to confirm and extend our findings.

## Conclusions

The distribution of pediatric acute leukemia subtypes in our population is close to data published at local, regional, and international levels. This study confirms the value of flow cytometry as a tool for lineage assignment and diagnostic support in pediatric acute leukemias, contributing to the classification of ALL, identification of acute myeloid leukemia with minimal differentiation, and diagnosis of BAL in a resource‑limited setting. The high frequency of aberrant antigen expression observed in our cohort underscores the need for combined analysis of several markers to avoid interpretation errors. These findings justify the adoption of a standardized diagnostic algorithm that systematically combines cytology, cytochemistry, and immunophenotyping. However, we acknowledge that the absence of cytogenetic and molecular data prevents a complete classification according to the WHO 5th edition and limits prognostic interpretation. Prospective multicenter studies including cytogenetics, molecular analyses, and patient follow‑up are necessary to clarify the prognostic value of the described profiles and to improve the management of pediatric acute leukemias in Morocco.
